# Accrual and Dismemberment of Brain Tumours Using Fuzzy Interface and Grey Textures for Image Disproportion

**DOI:** 10.1155/2022/2609387

**Published:** 2022-07-30

**Authors:** Pravin R. Kshirsagar, Hariprasath Manoharan, V Siva Nagaraju, Hamed Alqahtani, Quadri Noorulhasan, Saiful Islam, M. Thangamani, Varsha Sahni, Amsalu Gosu Adigo

**Affiliations:** ^1^Department of Artificial Intelligence, G.H Raisoni College of Engineering, Nagpur, India; ^2^Department of Electronics and Communication Engineering, Panimalar Engineering College, Poonamallee, Chennai, India; ^3^ECE Department, Institute of Aeronautical Engineering, Hyderabad, India; ^4^King Khalid University, College of Computer Science, Center of Artificial Intelligence, Unit of Cybersecurity, Abha, Saudi Arabia; ^5^College of Computer Science, King Khalid University, Abha 61413, Saudi Arabia; ^6^Civil Engineering Department, College of Engineering, King Khalid University, Abha 61421, Asir, Saudi Arabia; ^7^Department of Information Technology, Kongu Engineering College, Perundurai, Tamilnadu, India; ^8^Lovely Professional University, Phagwara, Punjab, India; ^9^Center of Excellence for Bioprocess and Biotechnology, Department of Chemical Engineering College of Biological and Chemical Engineering, Addis Ababa Science and Technology University, Addis Ababa, Ethiopia

## Abstract

A neurological disorder is a problem with the neural system of the body, as a brain tumor is one of the deadliest neurological conditions and it requires an early and effective detection procedure. The existing detection and diagnosis methods for image evaluation are based on the judgment of the radiologist and neurospecialist, where a risk of human mistakes can be found. Therefore, a new flanged method and methodology for detecting brain tumors using magnetic resonance imaging and the artificial neural network (ANN) technique are applied. The research is based on an artificial neural network-based behavioral examination of neurological disorders. In this study, an artificial neural network is used to detect a brain tumor as early as possible. The current work develops an effective approach for detecting cancer from a given brain MRI and recognizing the retrieved data for further use. To obtain the desired result, the following three procedures are used: preprocessing, feature extraction, training, and detection or classification. A Gaussian filter is also incorporated to eliminate noise from the image, and for texture feature extraction, GLCM is considered in this study. Further entropy, contrast, energy, homogeneity, and other GLCM texture properties of tumor categorization are measured using the ANFIS approach, which determines if the tumor is normal, benign, or malignant. Future research will focus on applying advanced texture analysis to classify brain tumors into distinct classes in order to improve the accuracy of brain tumor diagnosis. In the future, MRI brain imaging will be used to classify metastatic brain tumors.

## 1. Introduction: Brain Tumors for Classification

One of the most serious neurological illnesses is a brain tumor. A brain tumor occurs when the cells in the brain grow abnormally and uncontrollably [1]. A benign brain tumor and a malignant brain tumor are designated as two elementary procedures of brain tumors. If no cancer is found, then brain tumors are known as benign tumors, while brain tumours are referred to as malignant tumours when malignancy is present. The first sort of cancerous or malignant brain tumors is a primary brain tumor, whereas the second type is a secondary brain tumor. The tumors that begin in the brain are benign tumors, whereas metastatic cancers spread from another location. The growth of a benign tumors is substantially slower than that of a cancerous tumor. The least aggressive tumors are benign tumors, and the most aggressive tumors are malignant tumors[2]. The threat level is determined by a combination of characteristics, such as the type of tumors, their location, their size, and their stage of growth [3]. Tumors might be seen in the central spinal canal or inside the cranium. According to the National Brain Tumor Foundation, there are 29,000 individuals identified and diagnosed with initial brain tumors in the United States of America each year. A total of over 13000 individuals are killed. Every year, 4200 people in the United Kingdom are diagnosed with brain tumors (estimate from 2007) [4]. Magnetic resonance imaging is one of the most effective tools for diagnosing brain tumors today. More detailed images are produced by this magnetic resonance image. The automatic detection of flaws in magnetic resonance images is very beneficial in a variety of diagnostic and therapeutic applications [4]. Medical practitioners and academics can investigate the brain using magnetic resonance imaging by gazing at it noninvasively. Now that digital image processing has advanced, radiologists can use automated approaches such as computer aided systems and artificial neural networks to increase their performance. The goal of the computer-assisted system is to improve the technique's predictive values by pre-reading medical images in order to show where anomalies are located and assess their characteristics [4].

One of the applications of artificial intelligence is the artificial neural network that mimics a biological neural network. The neuronal structure of the brain is used to create artificial neural networks [5] as a case of gaining knowledge through experience. An artificial neural network is made up of a series of interconnected neurons arranged in layers. The processing element is referred to as the neuron (PE). Each neuron receives input (processing element). The processing element processes the given input before delivering a single output. An artificial neural network [2] is a data processing system with interconnected components that functions similarly to a neuron. A powerful tool in the biomedical field is the artificial neural network. It can represent and capture complex input/output relationships. A neural network is a type of artificial intelligence that can be utilized in a variety of applications. In medicine, neural networks are frequently employed in grouping problems, such as pattern recognition. In our research, we deploy an artificial neural network to detect brain tumors. The neural network's main feature is that it is trained to learn by changing data frequently [6]. Grey level cooccurrence matrix is used in this study to extract features. The cooccurrence matrix for grey levels is highly useful for extracting texture features. Haralick is the one who introduces GLCM. For feature analysis, it provides a typical value. The most popular second order statistic is the GLCM. It is a method for extracting second-order statistical texture features [7]. The classification is done with a neurofuzzy classifier.

### 1.1. Literature Survey

An unsupervised self-organized map (SOM) technique is employed to classify MRI brain images in [8]. They used independent component analysis to minimize the dimensionality of the applied images in this work, which preprocessed MRI scans. With the use of a self-organizing map and the k-means clustering technique, the image was finally identified as normal or abnormal. A graphical user interface has been created that displays self-organizing mapped and clustered output and determines the image's abnormality area. This procedure is more accurate than the previous one. This method achieves a 98.6 percent accuracy, which is considerably superior to previous categorization techniques. Reference [9] designed and evaluated an enhancement and segmentation method based on a model of a pulse linked neural network and back propagation network using MR brain pictures. This innovative use of PCNN and BPN leads to improved image segmentation. The PCNN can definitely be employed as an image analysis tool, as demonstrated in this study. Reference [10] suggested an approach for detecting brain tumors that included preprocessing, gab our feature extraction, and back propagation network classification. The categorization accuracy in this experiment was found to be 89.9%. Different photos can be used to test the system. It is critical to employ a big amount of patient data in order to improve the system's accuracy. Vector quantization for learning, feature self-organizing mapping procedures, image segmentation, and fixed wavelet changes are mentioned in [11]. MRI is used to segment intelligent images into strong materials like GM, WM, and CSF, as well as pathological tissues such as tumors and edema.

An algorithm that associates inception and morphological procedures is created to remove the parts of the skull that are not relevant to this study. SWT was used to break down pictures into sub-bands. They apply spatial filtering algorithms to these sub-bands in order to generate a feature vector that will be fed into the SOM. An unsupervised SOM network performs the segmentation process. The SOM's output neurons are calibrated using a supervised LVQ technique. The suggested model can fragment brain images with virtuous accurateness, according to statistical analysis of the experimental findings. Their overall process took 20 seconds for each MR volume and could segment white matter, grey matter, cerebrospinal fluid tumor, and edema from MR pictures. In [12], an automated technique for classifying MRI brain pictures with various clinical conditions is shown. Pathologists use microscopes to examine pathologic tissues to evaluate the degree of normalcy versus disease. This is a time-consuming and exhausting process. This article uses an automated classification system to classify abnormalities as benign or malignant. This research uses a feed forward neural network to implement a conceptually basic classification system. Rough set theory is used to calculate texture features. The suggested approach efficiently classifies brain tumor abnormalities. The suggested algorithm in [13] describes a method for detecting and identifying brain tumors in MR images. It is an approach that is based on contours. On the basis of gradient vector flow, the algorithm detects regions of interest. Most algorithms simply employ a single feature for segmentation, such as intensity or texture, but this technique uses both texture space and intensity value approaches to determine the correct image boundary.

Reference [14] describes a method for classifying tumors on brain imaging. The key goal is to use the best feature set to distinguish between distinct aberrant brain pictures. The classification of proton magnetic resonance spectroscopy pictures is done in this article. However, the classification accuracy varies depending on the data set, which is one of the method's shortcomings. According to the findings, the proposed hybrid technique is accurate, quick, and stable. Reference [15] uses an artificial neural network as a classifier to classify brain pictures. In comparison to other classifiers, it has a high classification efficiency. Sensitivity, specificity, and accuracy have all been enhanced. The proposed method is efficient in terms of computation and produces decent results. Future work will focus on extracting more characteristics and expanding the training dataset to increase classification accuracy. They used an artificial neural network to create an automated approach for detecting brain tumors in EEG signals [6, 13]. As an input, the suggested system uses an EEG signal with artifacts. The EEG signal is used to extract features of interest for brain tumor detection. With the feature collected from EEG signals, an ANN is used to train a feed forward back propagation neural network. Finally, the trained feed forward back propagation neural network detected the existence of a brain tumor in the test signal when it was given as a test input. The proposed system's efficiency in detecting brain tumors has been proved by the experimental results.

The identification of a brain tumor utilizing BPNN and PNN was successful in [16]. The entire system operates in two stages: first, training, and then, testing. The image processing technique, such as Otsu's thresholding approach, is used for training. In artificial neural networks, texture features are employed for training. Cooccurrence matrices are constructed at angles of 0, 45, 90, and 135 degrees, and GLCM features are retrieved from the matrices. The method described above effectively classifies tumor kinds in brain MRI images. The system's next goal is to use different approaches to improve the ANN design. In comparison to conventional classification and segmentation processes, the system has been intended to be more accurate and faster. The system described in [17, 18] proposes unique procedures for detecting brain tumors from EEG waveforms using wavelet characteristics and artificial neural networks. A separate training network for the brain tumor and the healthy waveform is being considered. The system's performance was tested on 50 different EEG images and found to have an identification accuracy of 90%. The findings of the experiments reveal that both brain tumor and healthy EEG images are appropriately recognized [19].

### 1.2. Problem Statement

The existing detection and diagnosis methods for image evaluation are based on the judgment of the radiologist and neuro-specialist, where a risk of human mistakes can be found. Therefore, a new flanged method and methodology for detecting brain tumors using magnetic resonance imaging and the artificial neural network (ANN) technique are applied.

### 1.3. Motivation of Proposed Work

The research is based on an artificial neural network-based behavioral examination of neurological disorders. An artificial neural network is used to detect the brain tumor in this case. This endeavor will assist us in utilizing technology for the greater good of society because a brain tumor is one of the deadliest neurological conditions, it is critical to discover and treat it as soon as possible. Currently, the procedures for detecting brain tumors are dependent on the decisions of neurologist and radiologist, which can lead to human mistake, which is dangerous and this is a highly time-consuming operation.

As a result, in this study, an artificial neural network is used to detect a brain tumor as early as possible. This effort will be beneficial to the hospital's doctors as detectors may be used to detect deterioration, protect patients from injury during treatment, and eventually enhance the patient's result. This work is useful in the field of biological early cancer diagnosis and it aids doctors in their efforts to diagnose a brain tumor [20, 21]. This technique could also be valuable in medical fields like computer-assisted diagnostics and mammography.

### 1.4. Objectives

The following are the major goals of this study, in which an artificial neural network is implemented to detect a brain tumor.

1. To use magnetic resonance imaging to detect brain tumors.
2. To improve accuracy and yield, an autonomous approach was employed to detect a brain tumor utilizing an artificial neural network.
3. To shorten the time it takes to discover a brain tumor.
4. To assist doctors and radiologists in detecting brain tumors, as well as to determine the extent to which artificial neural networks are useful in brain tumor identification.

## 2. System Model Classifications Using Grey Level

The most frequent method for extracting the texture feature is grey level concurrence matrices (GLCM). The GLCM is used to extract texture information from the image in this brain tumor identification procedure. The second order statistical information of nearby pixels of the image is contained in this grey level co-occurrence matrix [22]. Thus, GLCM is employed to determine textural qualities where a better understanding can be achieved on the image content. The geometric approach of the second order [7] texture traits or GLCM features was used to discriminate standard and anomalous brain tumors characteristics. Five diagonal matrices are produced in four different altitudinal locations, such as parallel, perpendicular, right crosswise, and left diagonal with coordinates located at 0-, 45-, 90-, and 135-degree units. The additional diagonal matrix [23] is formed by taking the average of the four previous matrices and a set of five grey level co-occurrence matrix features are derived for training purposes in various orientations. Thus, the polynomials for coordinate system can be formulated using equations (1)–(4) as follows:

(1) $$\begin{array}{c} P_{1}\left(\Delta_{i}\right)=1, \end{array}$$

(2) $$\begin{array}{c} P_{2}\left(\Delta_{i}\right)=\sum_{i=1}^{n}\Delta_{\text{in}}-\frac{1}{2}, \end{array}$$

(3) $$\begin{array}{c} P_{3}\left(\Delta_{i}\right)=\sum_{i=1}^{n}\Delta_{1}^{2}-\Delta_{\text{in}}+\frac{1}{6}, \end{array}$$

(4) $$\begin{array}{c} P_{4}\left(\Delta_{i}\right)=\sum_{i=1}^{n}\Delta_{1}^{3}-\frac{3}{2}\Delta_{2}+\frac{1}{2}\Delta_{\text{in}}. \end{array}$$

In all the abovementioned equations, consider *P* to be the *N∗N* cooccurrence matrix for each subpicture. It contains crucial information about the pixel's positions with the same grey level values. The number of rows and columns in a grey level cooccurrence matrix is the same as the number of grey levels. Thus, the peripheral probability matrix is framed using *a* and *b* coordinates as represented in the following equation:

(5) $$\begin{array}{c} P_{i}\left(a,b\right)=\left[\begin{array}{ccc} P_{1}\left(a\right) & \cdots & P_{1}\left(b\right)\\ \vdots & d_{\text{in}} & \vdots \\ P_{n}\left(b\right) & \cdots & P_{i}\left(a\right) \end{array}\right], \end{array}$$

where (*P*(*i*, *n|d*)) represents the relative frequency matrix element with two pixels [5] and *d*_in_ represents the distance between two pixels, which is the specific angle that specifies the direction.

The GLCM, which contains data about the picture's textural qualities, is used to extract a set of 14 texture features from the MRI brain image [24, 25]. Entropy, variance, homogeneity, angular second moment, average, correlation, contrast, mean of correlation, maximum probability, dissimilarity are the textural features, which will have standard deviation values with respect to probability distribution function, and it can be represented using the following equation:

(6) $$\begin{array}{c} \sigma_{A}^{2}=\sum_{i=1}^{n}{\left(P_{i}\left(a\right)-\gamma_{i}\left(a\right)\right)}^{2}, \end{array}$$

(7) $$\begin{array}{c} \sigma_{B}^{2}=\sum_{i=1}^{n}{\left(P_{n}\left(b\right)-\gamma_{n}\left(b\right)\right)}^{2}, \end{array}$$

where *γ*_*i*_(*a*) and *γ*_*n*_(*b*) denote the mean values of coordinate systems.

Since the standard deviation values are observed from diffused tumor cells, the mean values in equations (6) and (7) are subject to the following constraints using binary variables:

(8) $$\begin{array}{c} \gamma_{\text{in}}^{2}\left(a,b\right)=\left\{\begin{array}{cc} 0, & \text{if }i+n=P,\\ 1, & \text{otherwise.} \end{array}\right. \end{array}$$

Equation (8) indicates that if *in* is equal to the distribution values, then 0 will be represented for detecting signals. Both values are not equal to the distribution function if 1 is represented. Thus, signals are conceded for further operations that must satisfy the following integral property:

(9) $$\begin{array}{c} S_{i}\left(t\right)=\int_{0}^{1}\gamma_{i}\left(a\right)\gamma_{n}\left(b\right)dadb\\ ={\left(-1\right)}^{\left(i-1\right)}\frac{\left|a\right|\left|b\right|}{\left(a+b\right)!}. \end{array}$$

Equation (9) determines the integral function with quality factor for operational tumors detection that varies on every factorial term. As quality factor is determined, the contrast parameter must be described before the observation of difference movement, and it can be mathematically framed using the following equation:.

(10) $$\begin{array}{c} C_{i}=\sum_{i=1}^{n}v_{i}*\left(P_{i}\left(a\right)+P_{i}\left(b\right)\right), \end{array}$$

where *v*_*i*_ denotes the variation matrix with subjective functions.

Since uniformity characteristics of the images are present during detection of brain tumors, an inverse momentum function must be represented in mathematical form, as small influence will be made if any inhomogeneous characteristics of images are observed [26]. Thus, the uniformity equation is framed as follows:

(11) $$\begin{array}{c} u_{i}\left(t\right)=\sum_{i=1}^{n}\frac{1}{1+{\left(P_{a}-P_{b}\right)}^{2}}, \end{array}$$

where *P*_*a*_,  *P*_*b*_ represent the inverse probability matrix that is varied with respect to time periods.

Once grey level features are extracted in texture form, then presence of brain tumors will be classified using artificial neural network where the integration process is described in the subsequent section.

## 3. Optimization Classifiers Using ANN

**Figure 1:**
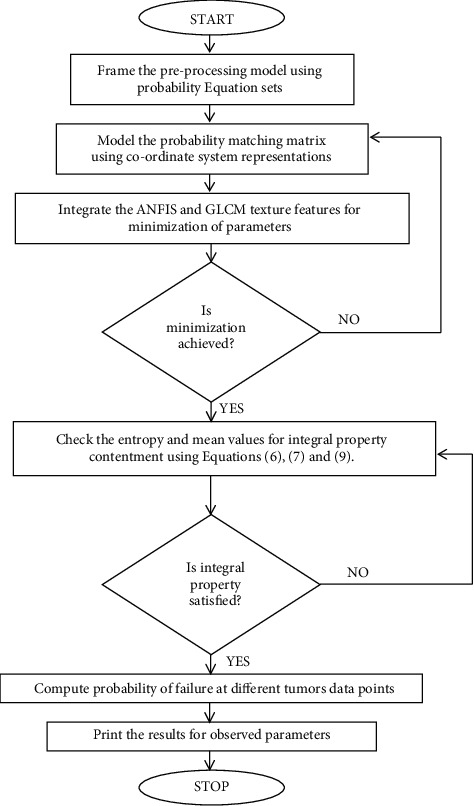
Design flow of integration.

To construct the neuro-fuzzy classifier, ANN and fuzzy (ANFIS) logic is integrated at the classification stage. Classification is a crucial step in determining the type of brain tumor and distinguishing between normal and abnormal brain tumors in Figure 1. Therefore, to classify the brain tumor images in this proposed research, ANFIS is employed. The neuro-fuzzy classifier is a hybrid of ANN and fuzzy logic, with the neural network determining the fuzzy system parameters [27, 28]. The neurofuzzy classifier is used in this study to detect anomalies in brain magnetic resonance imaging, where the hybrid intelligent system is the product of the neurofuzzy hybridization. Neuro-fuzzy hybridization is also known as a fuzzy neural network or a neurofuzzy system, and the fundamental benefit of this neurofuzzy system is that it is a universal approximator, which is essential with the full loading capacity to request interpretable If-Then rules [29].

The neurofuzzy system in general is based on a fuzzy system that is educated using a neural network-derived learning algorithm with few key steps, such as fuzzification, must be applied to the input physical variables where the degree of satisfaction will be calculated for available language phrases. After language phase measurement, conjugated fuzzy inferred parameters will be considered, and premise and defuzzification will be applied to the output. All of the above procedures are carried out in layers of neural networks that are placed in sequential order. The architecture of a neural network allows it to alter the weights in the form of extracted rule parameters. The abovementioned weight parameter follows a learning rule, which varies with motion constant, and it is expressed using the following equation:

(12) $$\begin{array}{c} w_{i}\left(n+1\right)=\rho \left(w_{n}+w\left(n-1\right)\right), \end{array}$$

where *w*_*n*_ and *w*(*n* − 1) represent the preceding and current weighting factor.

It is simple to design the activation function ANN, and this is termed as sigmoidal gain, which makes a smoothness restraint. In the proposed method, the sigmoidal function is spread out over a large range by lowering the scaling factor, thereby allowing training to route more quickly towards identified paths. Thus, the activation function is shown as follows:

(13) $$\begin{array}{c} Z_{i}\left(t\right)=\sum_{i=1}^{n}Z_{n}\left(e^{d_{\text{in}}^{2}/2\sigma_{a}}\right)/e^{d_{\text{in}}/2\sigma_{b}}, \end{array}$$

where *d*_in_ indicates the input distance of discrete image set.

The activation function, which is represented using exponential function, is subject to the following constraint:

(14) $$\begin{array}{c} {\text{activaion}}_{i}=\left\{\begin{array}{cc} d_{\text{in}}=0 & \text{if }e^{d_{\text{in}}^{2}/2\sigma_{a}}\rangle 1,\\ d_{\text{in}}=0 & \text{otherwise.} \end{array}\right\}. \end{array}$$

Each activation link has a weight and an integer number. Weights and integer numbers are utilized to govern the signal between two different neurons. Hence, there is no need to alter the weights if the network produces effective results. In case if the network produces undesirable results, the weights must be adjusted until the desired results are obtained. Thus, the error function for undesirable result case can be expressed using the following equation:

(15) $$\begin{array}{c} \vartheta_{i}=\sum_{i=1}^{n}\sqrt{Q_{\text{in}}\left(k\right)}*\delta^{2}, \end{array}$$

where *δ* represents the error function of *k* probability statistics.

## 4. Results and Discussion

The use of an artificial neural network to detect a brain tumor necessitates the acquisition of MRI images. Preprocessing MRI pictures, extracting features from MRI images using any appropriate method, feature selection is to improve system performance and classification using appropriate techniques. In this section, the outcomes of various processes that make up the design process will be discussed using the data set procedures. Reference data from many sources are collected for different classes of magnetic resonance pictures in order to develop the system for detecting brain tumors. As an outcome, these magnetic resonance scans are obtained from different sources where the magnetic resonance pictures are 256*∗*256 pixels in size. The software specification for classification process will be MATLAB image processing toolbox as clampdown points will be controlled in the simulation view. The design flow of the proposed method is classified using the following case studies.

1. Case study 1: classification of standard MRI images.
2. Case study 2: grouping for compassionate MRI images.
3. Case study 3: organization of menacing MRI images.

For all the above-mentioned case studies, both detection and segmentation will be made in stage 1, and the segmented images will be classified at final representations.

### 4.1. Case Study 1

**Figure 2:**
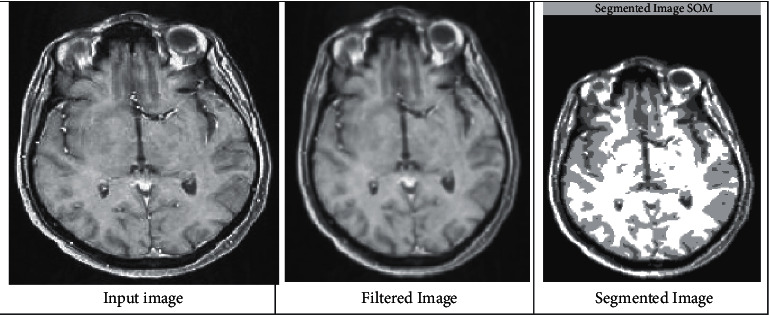
Segmentation of typical images.

The initial step in detecting a brain tumor is preprocessing the data. The preprocessing technique begins once the input MRI picture of a brain tumor has been acquired. It is much more difficult to remove undesired image distortion, enhance the image, and boost the contrast as it subsidizes to the system's increased accuracy. Image preprocessing in the projected model comprises of three phases such as filtering, segmentation, and retouching. All of these image enhancement processes are used to change the image brightness and grey level distributions. Therefore, image enhancement is utilized in the projected model to boost contrast between the normal brain and the cancerous region. To achieve the aforementioned outcome, a sharpening filter is introduced to observed the dissimilarity between the normal brain and the cancerous regions.  Figure 2 deliberates the normal MRI brain image that is segmented at various interval of time.

**Figure 3:**
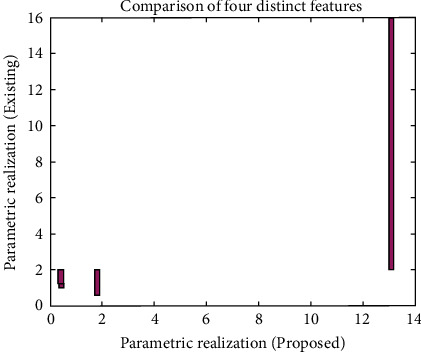
Parametric realizations.

For observing the values in Figure 2, a simulation analysis has been made, and different variable values, such as Autoc, contrast, energy, and entropy are plotted, and comparison with existing approaches [7] are made in Figure 3.

**Figure 4:**
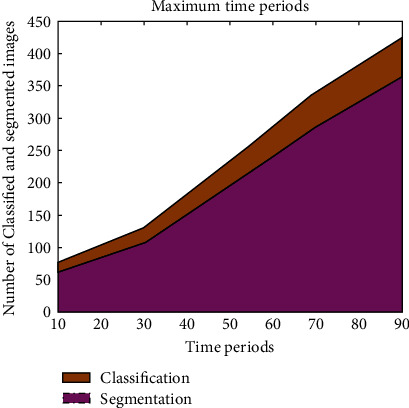
Maximum time period of standard image set.

From Figure 3, it can be realized that important parametric values, such as Autoc, energy, entropy, and contrast, are simulated using bar representations. Since the neural networks are employed, the values of Autoc, contrast, and energy are minimized as compared to existing approach [7]. However, the entropy of MRI images is maximized, thus leading to high segmentation within short time periods. This type of maximization is possible only when GLCM texture features are employed.  Figure 4 shows the corresponding time period representations of classification set.

### 4.2. Case Study 2

**Figure 5:**
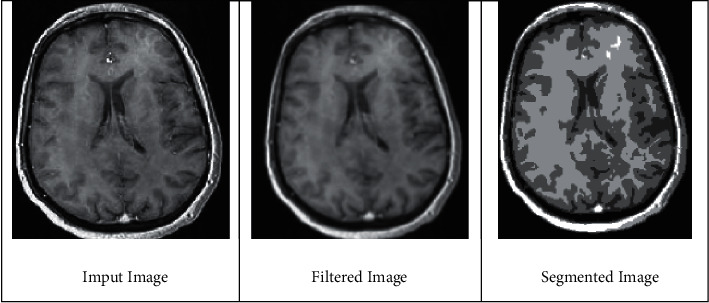
Segmentation of compassionate images.

It is extremely beneficial in many virtual applications that it is possible to distinguish several sections of the MRI images that resemble to the items of our interest and the expanses of the image that relates to the background process. Thresholding is a simple and convenient approach to separate an image based on dissimilar concentrations in both foreground and background. In the implementation procedure, the input is a grey scale image, and the output is a binary image that depicts segmentation, with black pixels corresponding to the background and white pixels corresponding to the foreground region of the image. If the implementation is up-front, then segmentation is achieved using a distinct parameter, which is termed as the concentration threshold. Each pixel in the image is compared to this threshold in a single pass, and the pixel is set to white in the output, which corresponds to the image's foreground region. If the intensity of the pixels exceeds the threshold, and if the pixel intensity is less than the threshold, the output sets the pixel to black, which corresponds to the image's background region.  Figure 5 shows the compassionate image set, which consists of the input image, which is varied from reference schematics, and the same filtered set is obtained in the next case, whereas the segmentation is achieved by comparing both input and filtered image set, and even MRI images for brain tumors are segmented with compassionate set regions.

**Figure 6:**
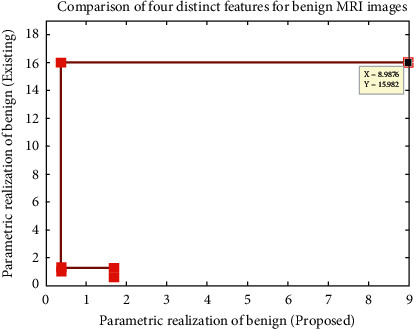
Parametric realizations of benign MRI images.

For the abovementioned illustration, values of parameters are realized using texture classifications and it is plotted in Figure 6. From Figure 6, the measurements indicate that even for benign images, values of Autoc, contrast, and energy are minimized but entropy values are maximized to a medium level of approach. Moreover, without ANFIS and GLCM, the existing model cannot be able to segment the benign images, thus a same set of values is repeated for this case study.

**Figure 7:**
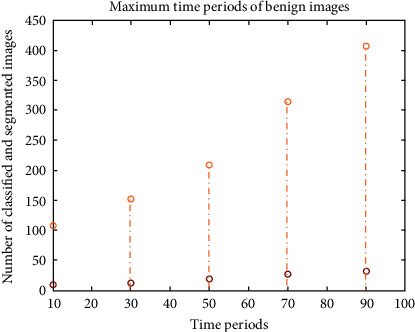
Time period of benign images.

The corresponding time period of calculation is represented in Figure 7, where the proposed method uses low implementation time for both classification and segmentation process.

### 4.3. Case Study 3

**Figure 8:**
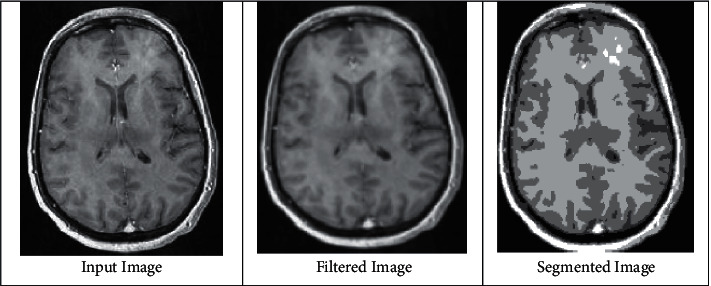
Segmentation of cancerous images.

The morphological operations in these case study techniques are image processing tools that are used to sharpen regions and fill gaps in binary images. The dilation procedure is used to fill up broken spaces at edges and maintain continuities at the boundary. MATLAB imdilate command is used to perform dilation operations where a 3*∗*3 square matrix structural element is employed to accomplish the dilation process. The region filled operation is used to fill the gaps in the tumor's region of interest morphological operators are employed to extract text regions in the proposed work. Vertical edges, horizontal edges, and diagonal edges are all mixed together in the text region.  Figure 8 shows the morphological representation of classification and segmented sets.

These vertical, horizontal, and diagonal edges are distributed individually in nontext regions as these three types of edges are mingled together in the text region and it can be determined where these three types of edges are found intermixed. To join isolated candidate text edges in each detail component sub-band of the binary picture, morphological dilation and erosion techniques are performed. Furthermore, commands are incorporated in the erosion and dilation operations in this morphological operation stage.

**Figure 9:**
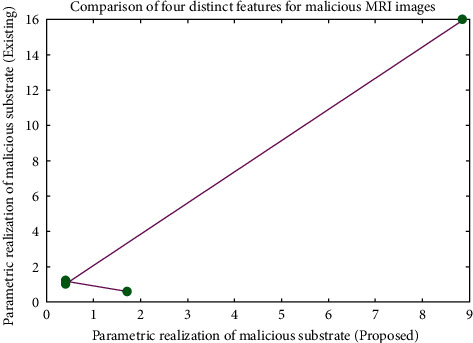
Parametric realizations of cancerous MRI images.

Figure 9 shows the parametric values of malicious MRI images, and in this case, an average value is achieved between standard and benign images for all four parametric determinations. However, no change is observed in the values of conventional techniques [7] due to deficiency of high filtering techniques using GLCM.

## 5. Conclusions

In the projected model, GLCM texture features and neurofuzzy (ANFIS) are used as classifiers to detect and classify MR brain images in this study. The process of introducing ANFIS indicates an effectual way of sensing magnetic resonance imaging (MRI) brain pictures into three different categories using a neuro-fuzzy classifier. The proposed method for classifying magnetic resonance images yields very promising results where the majority of available approaches can detect and classify magnetic resonance images into only two categories, such as normal and pathological. The proposed technique, on the other hand, uses text data to categorize MRI into the same three groups. The neuro-fuzzy can change its learning in real time, thanks to this speed of learning. This proposed model can also detect and classify MRI into three classes using MR brain images based on geometric texture features that not only replace traditional intrusive procedures but also help to reduce fatality rates.

### 5.1. Future Scope

Future work will focus on applying advanced texture analysis to separate brain tumors to enhance the exactness of brain tumor diagnosis. Later on, MRI brain imaging will be utilized to classify metastatic brain tumors.

## Data Availability

The datasets used and/or analyzed during the current study are available from the corresponding author on reasonable request.
